# The Treatment of Wounds by Iodoform Tampons

**Published:** 1888-02

**Authors:** 


### *THE TREATMENT OF WOUNDS BY IODOFORM TAMPONS.*

Dr. F. Bramann reports (*Archiv für klinische Chirurgie*, Berlin, 1887,) the results of treatment of wounds in Von Bergmann's clinic for some years past. The gauze employed is sterilized by means of steam at 212°, and, after drying, may be impregnated with an antiseptic solution. The sterilized gauze is used in cases of trifling operations in small wounds. In large wounds with more profuse secretion, it was thought best to obtain whatever advantage could be derived from the impregnation with corrosive sublimate, especially as the patients and operators are in the immediate vicinity of an audience coming direct from the anatomical rooms. The cotton employed is of late years merely sterilized. The towels, gum cloths, sponges, etc., are treated in a like manner. The silk used in sutures is wound on glass or metal spools, sterilized by steam, and enclosed in metal caskets. The catgut used for deep stitches (stitches of relaxation), and for ligatures, is kept ten to fourteen days in a solution of four parts bichloride, 800 of alcohol, 200 distilled water. This is frequently renewed. The catgut is then changed to an alcoholic sublimate solution of one to 800 alcohol and 200 parts of water, and is taken direct from this. The preparation of the patient consists in giving full baths, washing the region of operation with soap and water, shaving the part, rubbing the skin with ether, and disinfecting it with from 1 : 1,000 to 1 : 200 solution of sublimate. The instruments are kept in a three per cent. solution of carbolic acid. During the operation, the wound is often irrigated with 1 : 2,000 bichloride solution. In operations in the abdomen, the pleural cavity, the mouth, rectum and bladder, salicylic acid, 1 : 1,000, or boric acid, 1 : 200 is employed, and at the end of the operation a solution of iodoform in ether is generally used.

Next to strict antisepsis, the complete stoppage of bleeding is regarded as the chief agent in procuring union by first intention.

When the wound is dry, and the smallest bleeding vessels have been tied, the suture is applied with or without drainage, but only in those wounds which are considered absolutely antiseptic, and have not been infected through previous suppuration or contact with unclean materials. Among the cases treated in this manner are included all extirpations of tumors, removals of breasts, amputations, osteotomies, etc.

In wounds where the bleeding cannot be entirely stopped, the formation of a large clot is objectionable, not only on account of the pressure which it may make, as in fractures of the skull, but because of the risk of decomposition and blood poisoning. Although such clots may, through absorption and organization into connective tissue, aid in the process of repair, they sometimes remain fluid for long periods, and during that time are a source of danger. Therefore, when it is impossible to dry the wound absolutely, or where there is the least suspicion that it is not entirely aseptic, after thorough disinfection with 1 : 1,000 bichloride solution, and with an ethereal solution of iodoform applied to the wound by the means of a syringe, it is loosely packed with strips of iodoform gauze of several feet in length, and three to four inches broad. They are applied so that the larger part of each strip lies in the wound, and the ends come out at the angles. The sutures were formerly put in at this time, but this has been abandoned on account of the difficulty in keeping them disentangled, and of their adhesion to the iodoform gauze. The patient is now anæsthetized a second time for the application of the sutures. The tamponed wound is covered with sublimate gauze and cotton and an antiseptic bandage. If the secretions make their way through the dressings, the superficial layers are renewed, but the iodoform gauze is allowed to remain undisturbed for two days. If it is then removed by gentle fraction on the ends hanging out of the wound, the latter is found clean, unirritated, not reddened, absolutely dry, and it is only very exceptionally that a ligature is required. Careful suturing, with or without drainage, has resulted invariably in union by first intention, even in those cases in which, for any reason, as great weakness, or for the stoppage of bleeding from large vessels, the tampon has been left in from four to six days. His report of his results is extremely interesting, includes a large number of important cases, and appears to confirm his estimate of the value of this method.

